# ﻿Two new species of *Heteroconis* Enderlein, 1905 from China (Neuroptera, Coniopterygidae)

**DOI:** 10.3897/zookeys.1081.72606

**Published:** 2022-01-11

**Authors:** Yaru Zhao, György Sziráki, Zhiqi Liu

**Affiliations:** 1 School of Grain Science and Technology, Jiangsu University of Science and Technology, Zhenjiang 212004, China China Agricultural University Beijing China; 2 Department of Entomology, China Agricultural University, Beijing 100094, China Jiangsu University of Science and Technology Zhenjiang China; 3 Department of Zoology, Hungarian Natural History Museum, Baross utca 13, 1088 Budapest, Hungary Hungarian Natural History Museum Budapest Hungary

**Keywords:** Aleuropteryginae, dusty lacewings, identification key, morphology, taxonomy

## Abstract

Two new species, *Heteroconisyunnanensis***sp. nov.** and *Heteroconisorbicularis***sp. nov.**, are described from China. Both species differ from their congeners in characters of the male genitalia. *Heteroconisterminalis* (Banks, 1913) is redescribed based on examined Chinese specimens. A key to the adult males of *Heteroconis* from China is provided.

## ﻿Introduction

The genus *Heteroconis* was erected by Enderlein (1905) based on the type species *Heteroconisornate* Enderlein, 1905. This genus belongs to the subfamily Aleuropteryginae (Enderlein 1905). *Heteroconis* includes 61 species (Sziráki 2011; Oswald 2020) distributed in Australia, New Guinea, SE Asia and North Africa. *Heteroconis* is distinguished by the following characteristics: Antennae 17–18-segmented (or exceptionally 20 in *Heteroconistanzaniae* Meinander, 1998) and mostly bicolorous (whitish and blackish); male pedicel without ventral projections; stem of vein M with two thickened setae on fore wing; fore wing hind branch of vein RP fused with vein MA or connected with it by a cross vein; in hind wing, radial cross vein joining stem or fork of RP vein (Meinander 1972). The genus *Heteroconis* is divided into two species groups based on the position of the basal cross vein RP-M on the fore wing (Sziráki 2005). Hitherto, seven species of *Heteroconis* were known from China, belonging to the *Heteroconisterminalis* (Banks, 1913) group sensu Sziráki (2005). The two new species described in the present paper increase the number of *Heteroconis* species known from China to nine.

## ﻿Material and methods

All examined specimens were collected from China. The experimental methods were based on the methods of Zhao et al. (2021). All specimens examined in this study, including the holotypes of the new species, are deposited in the Entomological Museum of the China Agricultural University, Beijing (CAU). The abdomen was dissected from the body and macerated in a heated 5% KOH solution of for 5 minutes, then rinsed in water and 95% ethyl ethanol. Finally, the cleared abdomen was transferred to glycerol for dissection and study. After examination, the abdomen was placed in glycerol in a 200 μL microtube. The head and the thorax of the specimen are preserved in 95% ethyl alcohol in another 200 μL microtube. The two 200 μL microtubes were then placed in a 5 mL microtube at -20 °C. Wing vein terminology follows Breitkreuz et al. (2017). Genitalia terminology follows Meinander (1972), Sziráki (2001) and Sziráki (2002). Specimens were examined through an Optec SZ760 stereomicroscope. Photos were taken with a Nikon D5300 digital camera attached to a Leica DM2500 stereomicroscope, with further editing carried out in Adobe Photoshop CC 2018.

## ﻿Taxonomy

### ﻿Key to species of *Heteroconis* from China (males)

**Table:** 

1	Projections absent on the head (Fig. 1a)	**2**
–	Projections present on the head (Figs 3a, 5a)	**4**
2	Basal eight antennal segments pale (Meinander 1972: 88, fig. 43)	***H.picticornis***
–	Basal five or six antennal segments pale (Fig. 1a)	**3**
3	Sternite 9 with a ventral projection (Meinander 1972: 84, fig. 40)	***H.nigripalpis***
–	Sternite 9 without a ventral projection (Fig. 2)	***Heteroconisorbicularis* sp. nov.**
4	Frons with one projection (Liu et al. 2004: 367, figs 4–6)	**5**
–	Frons with two or three projections (Figs 3a, 5a)	**6**
5	Antennae with more than seven pale segments (Liu et al. 2004: 367, figs 4–6)	***H.electrina***
–	Antennae with no more than five pale segments (Liu et al. 2004: 370, figs 14–17)	***H.unicornis***
6	Frons with two projections (Fig. 3a)	**7**
–	Frons with three projections (Fig. 5a)	**8**
7	Last antennal segment pale (Fig. 3a)	***H.terminalis***
–	Last antennal segment dark (Liu et al. 2004: 368, figs 7–10)	***H.hainanica***
8	Last antennal segment pale (Fig. 5a)	***Heteroconisyunnanensis* sp. nov.**
–	Last antennal segment dark (Liu et al. 2004: 369, figs 11–13)	***H.tricornis***

#### ﻿ Heteroconis orbicularis sp. nov.

Taxon classificationAnimaliaNeuropteraConiopterygidae

03541857-D2E3-5B72-B635-8A36AA36187D

http://zoobank.org/B4C8916E-C887-45CD-B2D0-391DC480A665

Figures 1 , 2

##### Diagnosis.

Head projections absent; basal five or six flagellomeres pale; sternite 9 without a ventral projection.

##### Holotype.

Male, China, Yunnan Province, Jinghong City, Xishuangbanna Tropical Rainforest National Park, 22.0320°N, 100.8874°E, 25.iii.2019, leg. Yaru Zhao & Mingming Zou. ***Paratypes.*** 8 males, same data as holotype.

##### Description.

**Figure 1.**
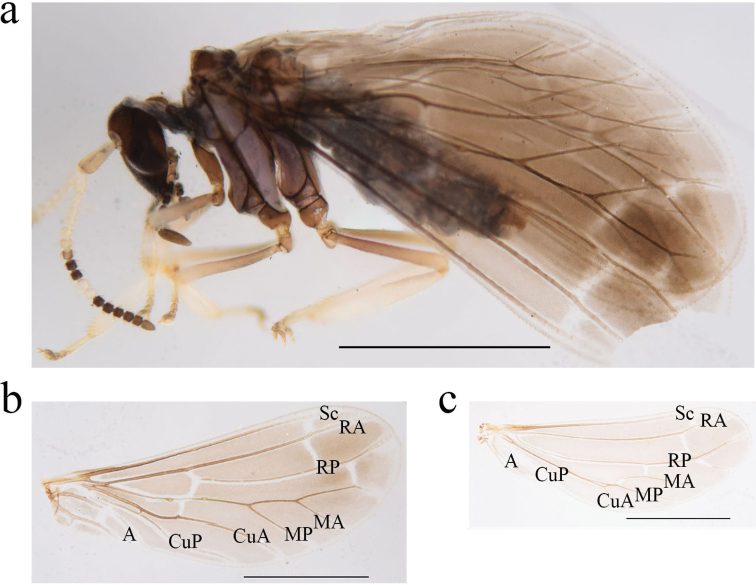
*Heteroconisorbicularis* sp. nov., holotype male **a** habitus, lateral view **b** head, dorsal view **c** forewing **d** hindwing. Scale bar: 1 mm.

**Male. *Head*** (Fig. 1a). Fuscous. Frons without projections. Eyes dark. Antennae 18-segmented. Basal six or seven as well as, eleventh and twelfth flagellomeres whitish, the others dark brown. Scape about 3-times as long as broad. Pedicel 2-times as long as broad. Maxillary and labial palps dark brown.

***Thorax*.** Fuscous, but thoracic sutures, apodemes, meso- and metanotum dorsal spots dark. Legs yellowish-brown.

***Wings*** (Fig. 1b–c). Fore wing membrane light grayish-brown. Basal cross veins RP-M and M-CuA meeting vein M between the median thickenings. Forewing length 2.8–3.1 mm, width 1.0–1.3 mm. Hind wing almost hyaline. Hindwing length 2.0–2.3 mm, width 0.8–1.1 mm.

**Figure 2.**
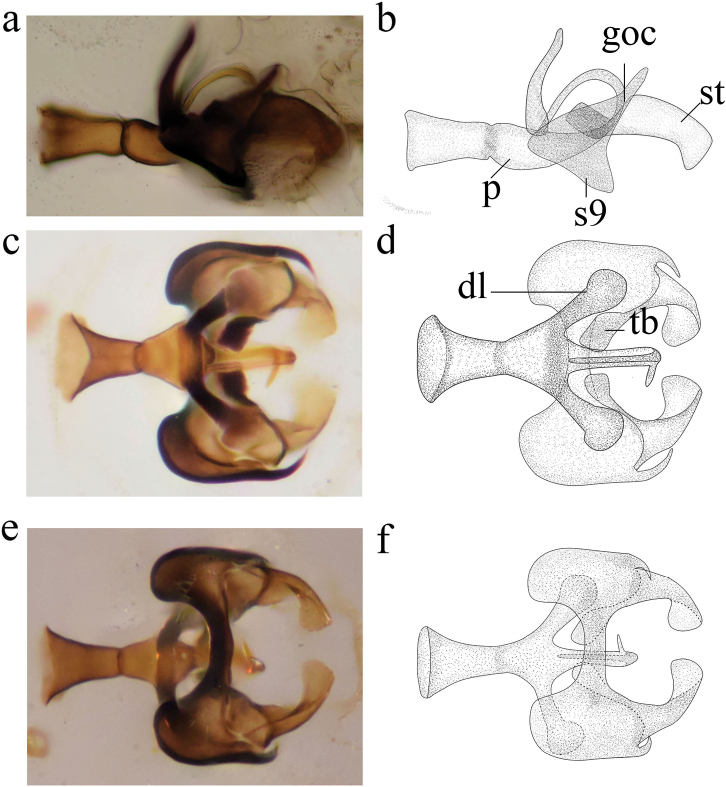
*Heteroconisorbicularis* sp. nov., holotype male, genitalia **a, b** lateral view **c, d** dorsal view **e** ventral view **f** penis, dorsal view. p, penis; s9, ninth sternite; tb, transverse band; goc, gonocoxite; dl, dorso-lateral projection of penis; st, stylus.

***Terminalia*** (Fig. 2a–d). Strongly sclerotized. Sternite 9 moderately narrow, without a ventral projection. Hypandrium absent, or probably fused with segment 9. Gonocoxites proximally wide, distally pointed and hooked. Stylus large and wide, median part hollow and connected to gonocoxites, caudal lobe with long inner setae (Fig. 2d). Distal part of penis curving upwards and forwards, then directed downwards.

**Female.** unknown.

##### Distribution.

China (Yunnan).

##### Remarks.

The new species belongs to the *Heteroconisterminalis* group sensu Sziráki (2005). *Heteroconisorbicularis* sp. nov. appears close to *Heteroconishelenae* Sziráki, 2001 and *Heteroconissakaeratica* Sziráki, 2002, based on the similarities of the male terminalia. However, there is no hypandrium in the new species. Moreover, flagellomeres 11–15 are brown in *H.helenae* and *H.sakaeratica*, whereas they are whitish in the new species. Furthermore, the penis of the new species is characterized by a dorsal projection on the apical part, absent in *H.helenae* and *H.sakaeratica*. *Heteroconisorbicularis* is distinguished by having its last two flagellomeres dark, by the absence of a hypandrium and a ventral projection on the 9^th^ sternite, by basal lobes of styli not connected, by distal part of penis forming a circle, and by dorsal projection of penis very long and slender.

##### Etymology.

The new species is named after its penis shape.

#### ﻿ Heteroconis terminalis

Taxon classificationAnimaliaNeuropteraConiopterygidae

(Banks, 1913)

0414E750-D6CD-5DA2-92F6-F075655832C1

Figures 3 , 4

Malacomyza terminalis Banks, 1913b: 220. Type locality: India (Maharashtra).

##### Material examined.

1 male, China, Guangxi Province, Baise City, Baise Revolt’s Memorial Hall, 23.9072°N, 106.6327°E, 6.iv.2019, leg. Yaru Zhao; 2 males, China: Guangxi Province, Pingxiang County, 22.1385°N, 106.8005°E, 12.v.1963, leg. Chikun Yang.

##### Remarks on the hitherto available descriptions of the species.

In the original description of *Heteroconisterminalis*, the male head is mentioned as having “a swollen cap or top piece” (Banks 1913). Withycombe (1925) re-examined a paratype of this species and gave a rather detailed description of its morphology, pointing out that “The latero-dorsal portions of the frons grow out as curved, horn-like projections”. Examination of the above Chinese specimens confirmed that there is a pair of latero-dorsal projections on the frons (Liu 2003; Liu et al. 2004). Wing measurements of the Chinese specimens examined in the present study are as follows: forewing length 2.8–3.2 mm, width 1.0–1.3 mm; hindwing length 2.6–3.0 mm, width 0.8–1.1 mm. Male terminalia sclerotized. Stylus about 5-times as long as broad in lateral view, with short and thick spines on distal third.

**Figure 3.**
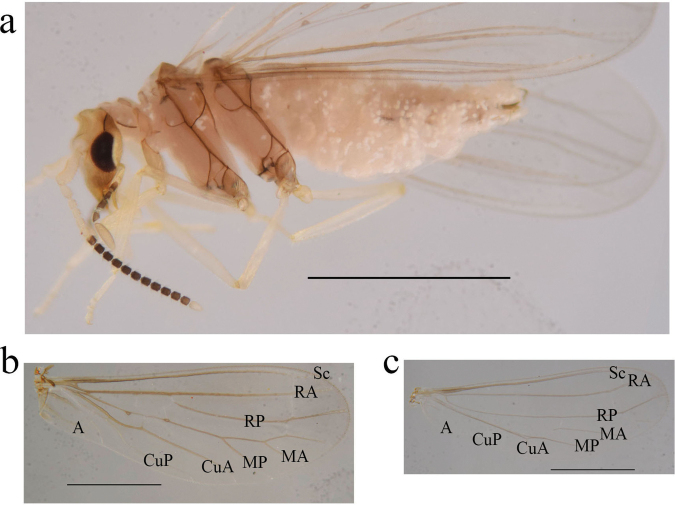
*Heteroconisterminalis* (Banks, 1913), male from Guangxi (China) **a** habitus, lateral view **b** head, dorsal view **c** forewing **d** hindwing. Scale bar: 1 mm.

**Figure 4.**
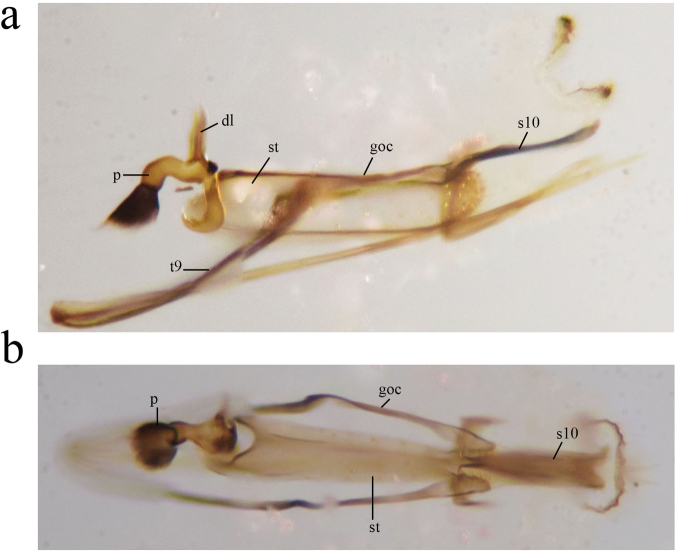
*Heteroconisterminalis* (Banks, 1913), Guangxi male, genitalia **a** lateral view. **b** dorsal view. p, penis; s10, tenth sternite; goc, gonocoxite; dl, dorso-lateral projection of penis; st, stylus; t9, ninth tergite.

##### Distribution.

China: Guangxi, Hainan, Yunnan; India: Bombay; Malaysia: Selangor.

#### ﻿ Heteroconis yunnanensis sp. nov.

Taxon classificationAnimaliaNeuropteraConiopterygidae

1FF3C4C9-E021-5823-9526-564EC1A1BC8A

http://zoobank.org/E9986666-63B4-431A-A283-6B64F476A732

Figures 5 , 6

##### Diagnosis.

Head with projections; basal eight flagellomeres pale; sternite 9 with ventral projection.

##### Holotype.

Male, China, Yunnan Province, Ruili County, Ruili Botanical Garden, 24.0723°N, 97.8174°E, 29.iii.2019, leg. Yaru Zhao.

##### Description.

**Figure 5.**
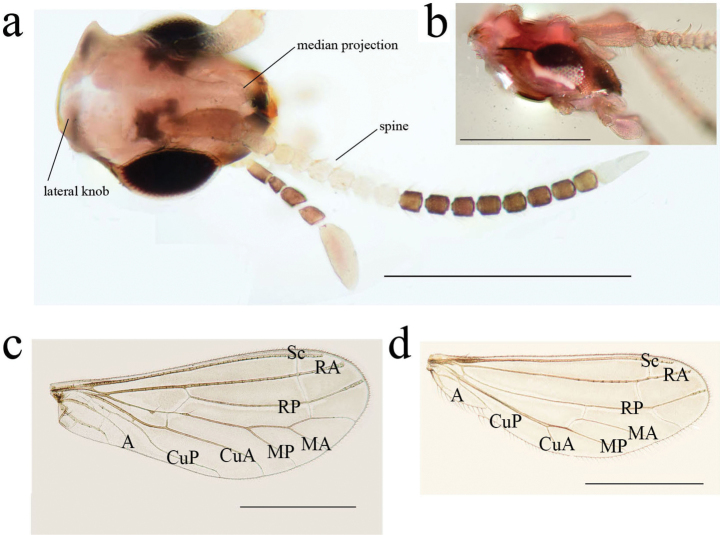
*Heteroconisyunnanensis* sp. nov., holotype male **a** habitus, lateral view **b** head, dorsal view **c** forewing **d** hindwing. Scale bar: 1 mm.

**Male. *Head*** (Fig. 5a, b). Fuscous. Frons with a pair of lateral knobs above antennae and a median projection between antennae. Eyes dark. Antennae 18-segmented. Basal eight and apical two flagellomeres whitish, the others dark brown. Both third and fourth flagellomeres with a short spine. Scape about 3-times as long as broad. Pedicel 2-times as long as broad. Four basal segments of maxillary palp dark brown, apical palpomere whitish. Labial palps dark brown.

***Thorax*.** Fuscous. Thoracic sutures, apodemes, meso- and metanotum dorsal spots dark. Legs yellowish brown.

***Wings*** (Fig. 5c, d). Fore wing membrane light grayish-brown. Forewing length 2.7 mm, width 1.2 mm. Hind wing almost hyaline. Hindwing length 2.2 mm, width 0.8 mm.

**Figure 6.**
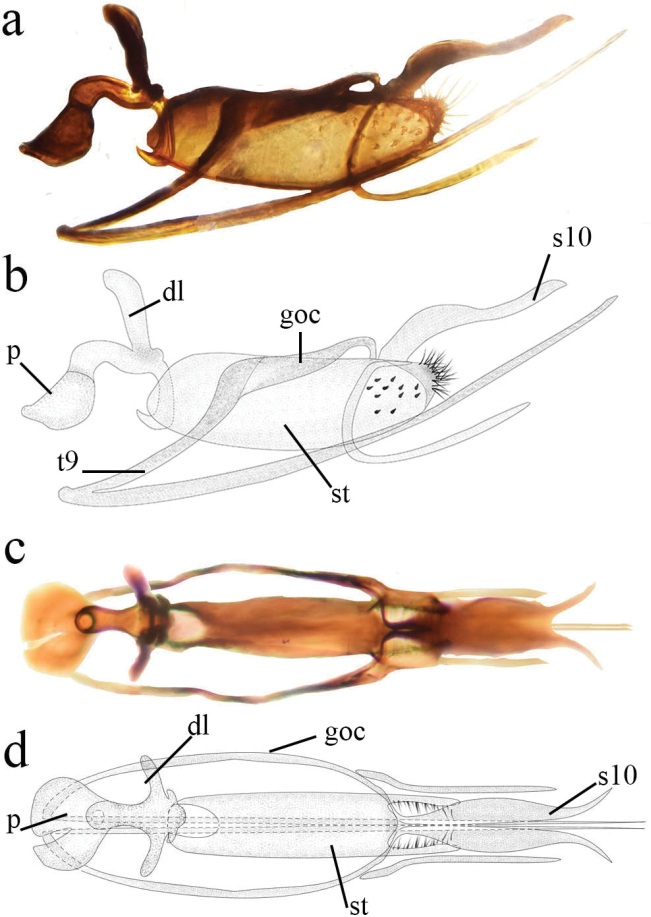
*Heteroconisyunnanensis* sp. nov., holotype male, genitalia **a, b** lateral view **c, d** dorsal view. p, penis; s10, tenth sternite; goc, gonocoxite; dl, dorso-lateral projection of penis; st, stylus; t9, ninth tergite.

***Terminalia*** (Fig. 6a–d). Sclerotized. Sternite 9 with a very long and slender ventral projection. Hypandrium absent, or probably fused with segment 9. Gonocoxite-stylus complex with long hairs on inner surface, caudal ending with long hairs on prominent bases. Stylus about 3-times as long as broad in lateral view, with short and thick spines on distal third. Sternite 10 elongated, slightly sinuous in lateral view, caudal part lyriform in dorsal view. Basal apodeme of penis moderately developed, dorso-lateral projection rather small. Tube of penis rather thick between the basal and dorso-lateral projections, distal part slender, directed downwards and forwards.

**Female.** unknown.

##### Distribution.

China (Yunnan).

##### Remarks.

The new species belongs to the *Heteroconisterminalis* group sensu Sziráki (2005). The male genitalia suggest a relationship with *Heteroconisterminalis*. However, the new species is characterized by the presence of a median projection on the male frons between the antennae, which is absent in *H.terminalis*. The two apical flagellomeres are whitish in the new species, whereas only the apical one is whitish in *H.terminalis*. Stylus about 3-times as long as wide in the new species, about 5-times as long as wide in *H.terminalis*. *Heteroconisyunnanensis* sp. nov. is distinguished by having the last two flagellomeres whitish, by the presence of a median projection between the antennae, by flagellomeres 3 and 4 with a short spine, and by stylus about 3 times as long as wide in lateral view.

##### Etymology.

The new species is named after its type locality.

## Supplementary Material

XML Treatment for Heteroconis orbicularis

XML Treatment for Heteroconis terminalis

XML Treatment for Heteroconis yunnanensis
